# Efficacy of levothyroxine on growth and development in children with congenital hypothyroidism: A meta-analysis

**DOI:** 10.1097/MD.0000000000041499

**Published:** 2025-02-21

**Authors:** Wenguan Liang, Lin Tu

**Affiliations:** a Department of Child Healthcare, The Fifth People’s Hospital of ZhongShan, ZhongShan, China.

**Keywords:** congenital hypothyroidism, growth and development, levothyroxine, META

## Abstract

**Background:**

The aim of this study is to systematically assess the efficacy of levothyroxine (L-T4) in the treatment of growth and development among children diagnosed with congenital hypothyroidism (CH) through meta-analysis, with the ultimate goal to provide clinicians with a more robust and effective evidence-based foundation for treatment decisions.

**Methods:**

We conducted a comprehensive search of relevant literature from both domestic and international resources, and meticulously screened out clinical studies which meet our inclusion criteria, that is without any time restrictions and the deadline was September 25, 2024, and the language was limited to English and Chinese only. Subsequently, we integrated and analyzed the data using standard meta-analysis methodologies under the Preferred Reporting Items for Systematic reviews and Meta-Analyses rules, aiming to derive more precise and reliable conclusions by summarizing and comparing the findings of multiple studies.

**Results:**

A total of 17 studies with 5 diagnostic indexes of IQ, height, weight, head circumference, and bone age were included, and these studies collectively reported on 1934 patients, with 951 and 983 patients, respectively being divided into the experimental and control group. The included studies were of high quality, and the results of our meta-analysis showed that L-T4 treatment could effectively promote the physical development of children with CH. Specifically, children in the experimental group had a higher level of IQ [mean difference [MD] = 8.38, 95% confidence intervals [CI] (6.89, 987)], height [MD = 6.71, 95% CI (6.08, 7.35)], weight [MD = 1.31, 95% CI (1.14, 1.49)], head circumference [MD = 3.83, 95% CI (3.52, 4.13)], and bone age [OR = 3.49, 95% CI (2.15, 5.68)] compared to those in the control group.

**Conclusion:**

L-T4 is an effective drug for the treatment with CH in children, which significantly promote their growth and development while improving thyroid function. This finding provides strong evidence and support for clinicians, contributing to the progress and advancement in the field of CH treatment.

## 1. Introduction

Congenital hypothyroidism (CH) is a common pediatric endocrine disorder characterized by hypothalamic–pituitary–thyroid axis dysfunction in newborns,^[1]^ resulting in inadequate thyroid hormones (THs) production or varying degrees of deficiencies from mild to severe.^[2]^ This condition can arise from multiple factors, including hypoplasia, congenital agenesis, and ectopic thyroid.^[3]^ If children are not treated promptly after birth, CH will lead to both growth and mental retardation, causing a serious burden on families and society.^[4]^ According to the statistics, the prevalence of CH varies from different regions of the world, with some studies reporting rates in newborns of about 1/4000.^[5]^ The prevalence is approximately 1/1200 in Asian Indian infants, 1/2380 in Asian (Chinese and Vietnamese) infants, 1/1600 in Hispanic infants, 1/3533 in non-Hispanic White infants, and 1/11,000 in non-Hispanic Black infants.^[6]^ In addition, results from newborn screening programs indicate an increasing trend in the prevalence of congenital primary hypothyroidism, which may stem from the lowered threshold of thyroid stimulating hormone (TSH) at screening and the detection of thyroid in situ.^[7]^ Early diagnosis and treatment of CH are crucial, as untreated CH can lead to intellectual and physical developmental disorders in childhood.^[8]^

Levothyroxine (L-T4) is the preferred treatment for CH, and usually considered as positive for its efficacy on children’s growth and development^[9]^; however, there still exists some uncertainties about issues such as L-T4 dosage, treatment timing, and long-term effects.^[10]^ And studies on the optimal L-T4 dose, particularly the initial dose, show variability, with some supporting the use of higher starting doses (>10 μg/kg) and others suggesting that lower doses may be equally effective.^[11]^ Early intervention is essential for the prevention of mental retardation, but further research is needed to determine the optimal start time and the duration of treatment. Moreover, although L-T4 treatment can improve growth indicators, its effects on children’s long-term neuro-development are still not fully understood, and some studies suggest that some children may still suffer from learning disabilities or other neuro-developmental problems despite L-T4 therapy.^[12]^

Therefore, this paper aims to put forward a comprehensive assessment of the L-T4 efficacy in promoting the growth and development of children with CH under the guidance of a systematic literature review and meta-analysis. By doing so, we aim to provide clinicians with more reliable evidence to guide rational medication use and individualized treatment plans for children with CH. Meanwhile, our findings will offer further research orientations for the future, thus, promoting the continuous progress and advancement in the field of CH treatment.

## 2. Information and methods

This meta-analysis was conducted and written in accordance with the Preferred Reporting Items for Systematic reviews and Meta-Analyses. The protocol was not registered.

### 2.1. Literature search

Keywords were identified related to the topic of this study, including “levothyroxine,” “congenital hypothyroidism,” “growth and development,” and “therapeutic efficacy.” In order to collect relevant literature as comprehensively as possible, PubMed, Cochrane Library, Embase, and Chinese databases such as China Knowledge Network and Wanfang Database were searched. There was no time limit for literature search and the deadline was September 25, 2024, but the language was limited to English and Chinese. After identifying the keywords and databases, a specific search formula is developed, which combined subject words, free words, and Boolean operators to ensure the accuracy and comprehensiveness of the search results. The specific search terms and search formulas are shown in Table 1.

**Table 1:** Literature search strategy.

Category	Chinese search terms	English search terms
Disease	Congenital hypothyroidism	Congenital hypothyroidism
Treat	levothyroxine	Levothyroxine
Growth and development	Growth and development, height, weight, head circumference, IQ	Growth and development, height, weight, head circumference, IQ
Retrieval based	“Levothyroxine” AND “Congenital Hypothyroidism” AND (“Growth and Development” OR “Efficacy”)	(Levothyroxine OR L-T4) AND (Congenital Hypothyroidism OR CH) AND (Growth AND Development OR Effectiveness OR Therapeutic Effect)

To comprehensively collect relevant literature on the topic of this study, which focuses on the therapeutic efficacy of L-T4 in the growth and development of children with CH, several key databases were selected, including PubMed, Cochrane Library, Embase, as well as Chinese databases such as China Knowledge Network and Wanfang Database. The search was conducted without a time limit but was limited to English and Chinese language articles. Keywords related to the study topic included “levothyroxine,” “congenital hypothyroidism,” “growth and development,” “therapeutic efficacy,” and others. A specific search formula was developed to ensure the accuracy and comprehensiveness of the search results, combining subject words, free words, and Boolean operators.

Literature inclusion criteria: (a) the included studies were clinical trials, observational studies, or randomized controlled trials; (b) they explicitly targeted children with CH; (b) the intervention in the study was L-T4 treatment; (c) the outcome indicators should contain assessment indicators about the growth and development of the children, such as height, weight, and intellectual development, and should be able to provide sufficient data for analysis. (d) The included literature should be peer-reviewed and published in high-quality academic journals to ensure the reliability and validity of the study results.

Literature exclusion criteria: (a) studies not related to the growth and development of children with CH treated with L-T4; (b) non-original research literature such as reviews, commentaries, conference reports; (c) multiple repetitive publications or overlapping data for the same study; (d) non-Chinese and English literature.

### 2.2. Literature screening and data extraction

The process of literature screening and data extraction was independently conducted by 2 researchers with cross-verification performed at every stage to ensure consistency and accuracy. The literature extraction process included several key elements: (1) basic information of the included literature, such as study title, first author, publication time, etc; (2) patient grouping information of the included literature, including male and female ratio, number of people in each group, and age distribution; (3) specific interventions of the randomized controlled trial (RCT) along with entire treatment duration; (4) information on the narrative of risk of bias evaluation; (5) summarization of the outcome indexes and data. The Cochrane Evaluation Handbook was used to evaluate the methodological quality of the included literature for RCTs.

Two researchers independently conducted the literature screening, starting with an initial screening of the literature based on predetermined inclusion and exclusion criteria, which included reviewing the titles and abstracts of the literature to determine compliance with the topic and purpose of the study. Throughout the screening process, the 2 researchers cross-verified their findings to ensure the consistency and accuracy during the screening process.

After identifying the included studies, 2 researchers independently extracted the required information from each included literature. Data extraction included (a) basic information, such as the study title, first author, and publication date; (b) information about the study patient subgroups; and (c) the specific interventions of the RCT; the Narrative information related to risk of bias evaluation, methodological quality evaluation of RCTs using the Cochrane Evaluation Handbook for the included literature, outcome indicators and data summarization, including data on major outcome indicators such as growth and development. Once data extraction was completed, the 2 researchers cross-checked meticulously to ensure the consistency and accuracy of the extracted data.

### 2.3. Statistical analyses

In the process of literature screening, the EndNote X7 Literature Manager facilitated the extraction of key information, including authors, literature titles, and import of literature abstracts. Other software like EXCEL and Word were also applied to the initial extraction and analysis of the information from screened literature in order to find out its characteristics and to make adequate preparation for the subsequent research projects.

Revman 5.3 software was used for the combination of effective sizes in the included literature, and relative risk was evaluated for dichotomous information. When measurements and units were consistent, weighted mean difference (MD) should be used for continuous variables, that is, metrics; but when units were inconsistent, standardized mean difference was applied accordingly. And all analyses were based on 95% confidence intervals (CIs).

Heterogeneity analyses were performed on the included studies. Heterogeneity between studies was indicated if the heterogeneity test showed *P* < .1 or I² > 50%. In such cases, subgroup analyses as well as sensitivity analyses should be used to explore the factors that contributes to heterogeneity from a clinical perspective to the maximum extent as possible. If the source of heterogeneity remained unclarified, a random-effects model would be the option for analysis. Conversely, if *P* > .1 or I² < 50% indicating homogeneity among the study literature, a fixed-effects model was expected to be selected.

For outcome indicators with more than 9 included literature, funnel plot analysis can be used as an approach to assess the presence of publication bias.

## 3. Results

### 3.1. Results of literature inclusion

**Figure 1.**
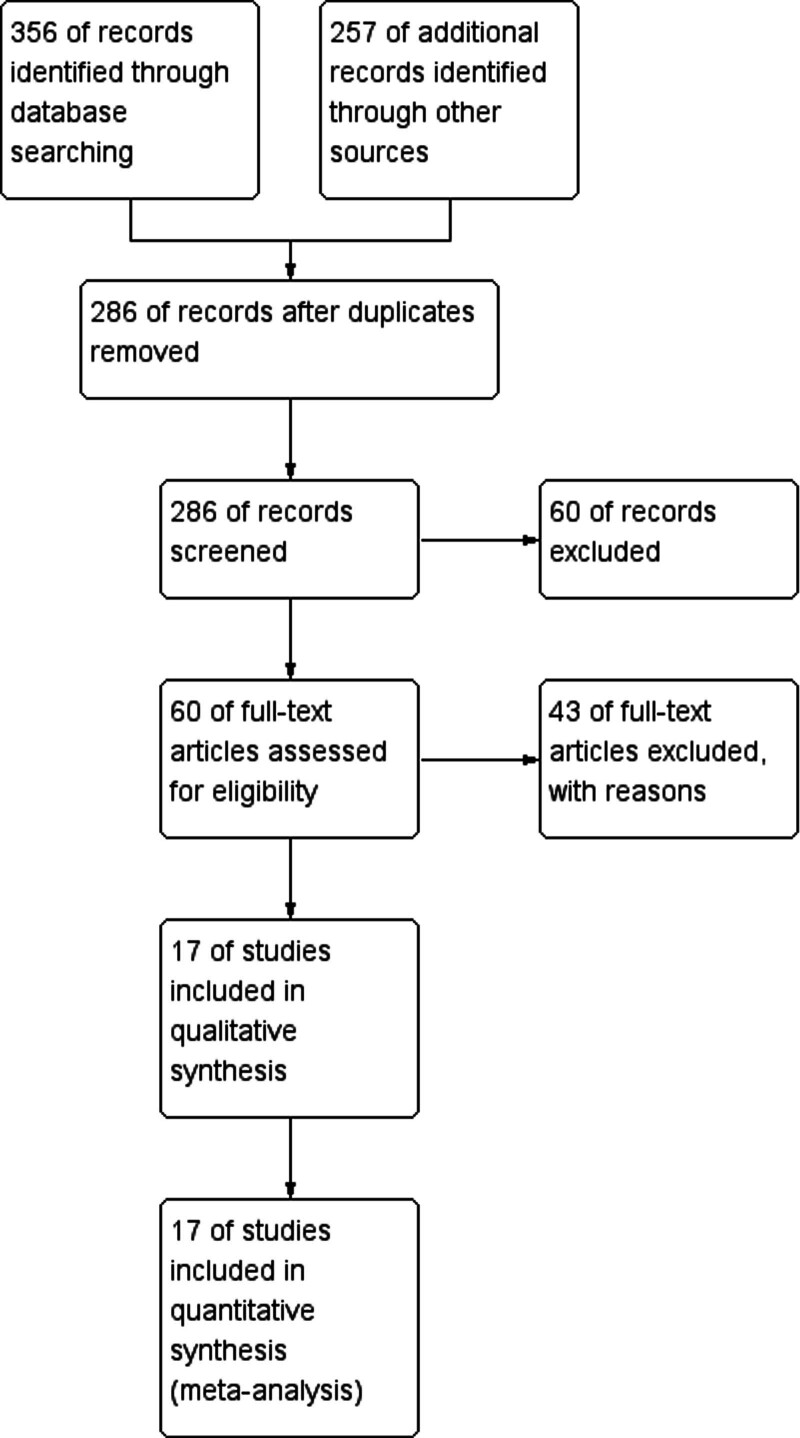
Literature inclusion process.

In this study, a total of 613 literatures were collected from various academic data platforms after database search. After removing duplicate literature, 286 articles were screened for initial screening. Further scrutiny, involving a review of titles and abstracts, led to the exclusion of 226 articles, primarily due to their features as reviews, irrelevance to the study topic, or focus on animal studies. Subsequently, an in-depth screening of the full text of the remaining 60 literatures was conducted. Based on criteria such as study quality, methodology, and outcome metrics, an additional 43 noncompliant literatures were considered as noncompliant and excluded. Ultimately, 17 high-quality studies closely related to the topic were successfully included in the analysis. As shown in Figure 1.

#### 3.1.1. General characteristics of the literature

A total of 17 studies were included, 6 studies were comparative studies of treatment with L-T4 versus no treatment, and 11 were comparative studies of dose and duration of use, and the outcome metrics included at least 1 of IQ, height, weight, head circumference, and bone age. Thousand nine hundred thirty-four patients were included, 951 and 983 in the trial control group, respectively See table 2 for details. After screening, a total of 17 literatures^[13–39]^ were finally included in the study.

**Table 2:** General characteristics of the included literature.

Name and surname	Number of cases in the observation group	Number of cases in the control group	Pilot group methodology	Control group method	Outcome indicator
Bongers-Schokking JJ (2000)^[13]^	34	27	<9.5 µg/kg/d	≥9.5 µg/kg/d	1
Bülbül M (2009)^[14]^	40	37	Levothyroxine therapy	–	2, 3
Casado de Frías E (1993)^[15]^	25	25	Levothyroxine therapy	–	2, 5
Dubuis JM (1996)^[16]^	22	23	Levothyroxine therapy	–	1
Esposito A (2022)^[17]^	36	36	High (12.6–15 mg/kg/day)	Low (10–12.5 mg/kg/day)	1
Heyerdahl S (1991)^[18]^	54	46	Levothyroxine therapy	–	1
Jones JH (2008)^[19]^	99	152	50 mg/day	25 mg/day	2, 3, 4
Li S (2021)^[20]^	30	30	10.1–15 mg/kg/day	6–10 mg/kg/day	1, 2, 3, 4
Lu YH (2012)^[21]^	45	45	Treatment is started when the baby is <1 month old	Intervention for infants between 1 and 3 months	1, 2, 3
Meng Y (2014)^[22]^	93	75	30–90 day interventions	<30-day intervention	1, 2, 3
Niuro L (2023)^[23]^	44	59	10–15 mg/kg/day	6–8 mg/kg/day	2, 3, 4
Peroni E (2014)^[24]^	39	39	10–14.7 μg/kg/tablet	10–14.7 μg/kg/fluids	1
Pniewska-Siark B (2006)^[25]^	102	102	Levothyroxine therapy	–	1, 2, 5
Salerno M (2002)^[26]^	42	41	8.1–15 mg/kg/day	6–8 mg/kg/day	1
Weber G (1995)^[27]^	58	58	Levothyroxine therapy	–	5
Zhang M (2020)^[28]^	158	158	Levothyroxine therapy	–	5
Zheng LL (2022)^[29]^	30	30	1–5 μg/(kg·d)	6–10 μg/(kg·d)	1, 2, 3, 4

For diagnostic indicators, 1 IQ, 2 height, 3 weight, 4 head circumference, and 5 bone age.

#### 3.1.2. Evaluation of the quality of literature

**Figure 2.**
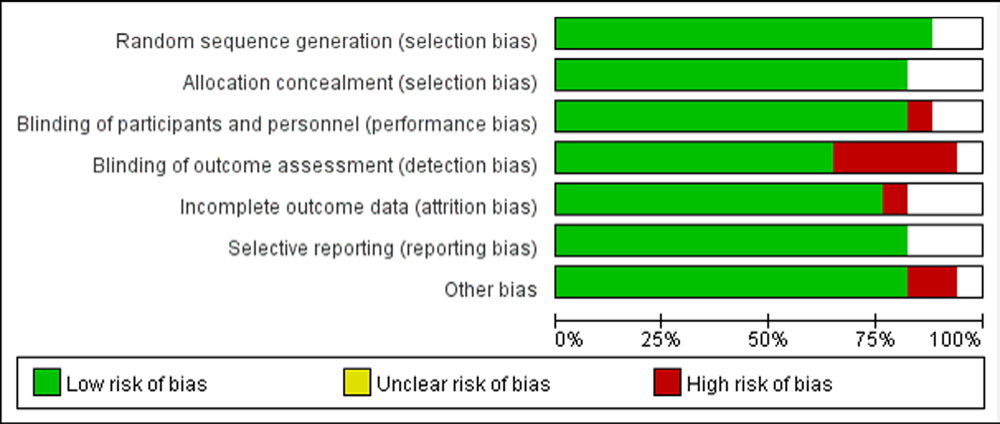
Literature quality assessment.

As shown in Figure 2, few of the included literature were high risk, and the overall quality of literature included in this study was high.

### 3.2. Meta-analysis results

#### 3.2.1. Meta-analysis results on IQ development

**Figure 3.**
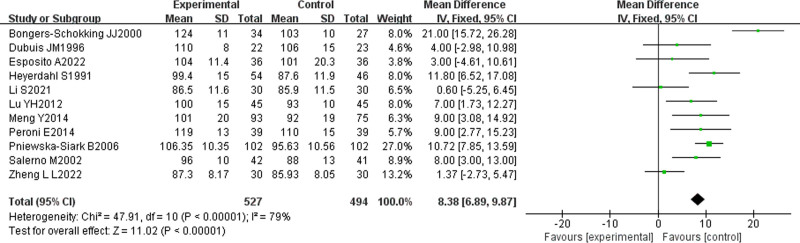
Meta-analysis results of IQ.

Eleven studies from the included literature^[13,16–18,20–22,24–26,29]^ examined the efficacy of L-T4 on IQ development in children with CH, involving a total of 1021 children, with 527 and 494 patients, respectively assigned to the treatment and control groups. Heterogeneity analysis with I² > 50%, random-effects model was chosen, and META-analysis showed a combined effect size of MD = 8.38, 95% CI (6.89, 987), Z = 11.02, *P* < .00001, indicating that IQ development of children in the experimental group was significantly better than that in the control group, with statistically significant differences. As shown in Figure 3.

**Figure 4.**
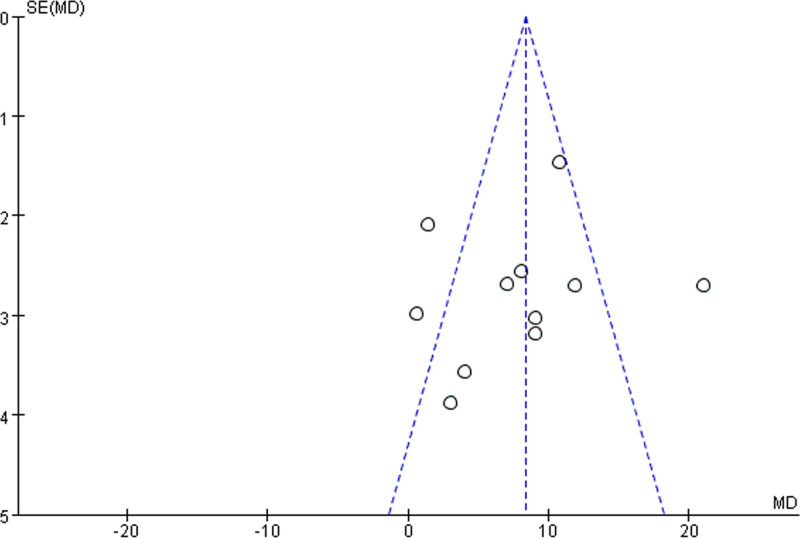
Funnel plot of IQ developmental efficacy.

The number of literature on the efficacy of L-T4 on IQ development of children with CH was ≥ 9. Further bias analysis was performed, and the funnel plot presented in Figure 4 shows that most of the literature were concentrated within the funnel. However, the distribution was not entirely symmetrical, suggesting that there was publication bias among the literature, albeit to a minor degree.

#### 3.2.2. Meta-analysis results on height development

**Figure 5.**
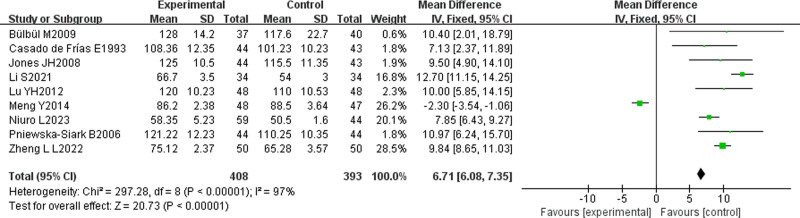
Meta-analysis results of height.

Nine studies in the included literature^[14,15,19–23,25,29]^ investigated the efficacy of L-T4 on height development in children with CH, involving a total of 801 children, with 408 and 393 patients, respectively assigned to the treatment and control groups, respectively. Heterogeneity analysis I² = 97% > 50%, random-effects model was selected, and META analysis showed a combined effect size MD = 6.71, 95% CI (6.08, 7.35), Z = 20.73, *P* < .00001, indicating that the height development of children in the experimental group was significantly better than that in the control group, and the difference was statistically significant. As shown in Figure 5.

**Figure 6.**
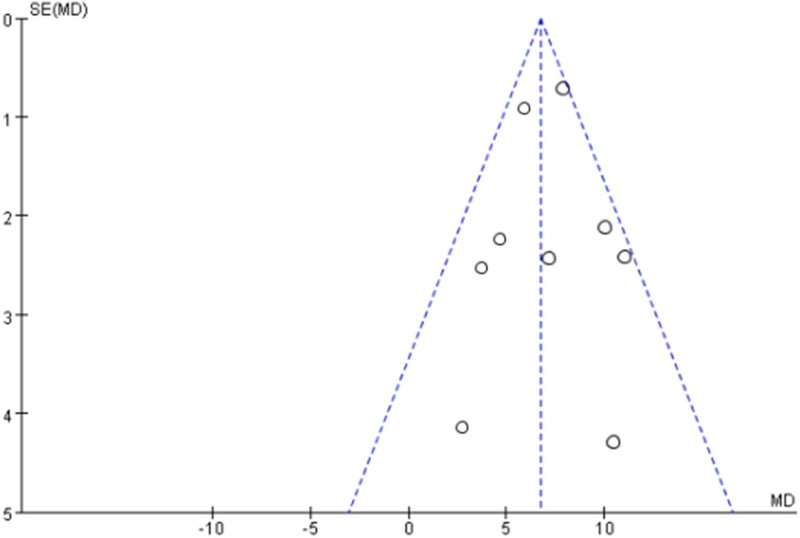
Funnel plot of IQ developmental efficacy.

The number of literature on the efficacy of L-T4 on height development of children with CH was ≥ 9. Further analysis of bias was performed, and the funnel plot is shown in Figure 6. The literature in the figure was concentrated within the funnel with symmetrical distribution, suggesting no publication bias among the literature.

#### 3.2.3. Meta-analysis results on weight development

**Figure 7.**
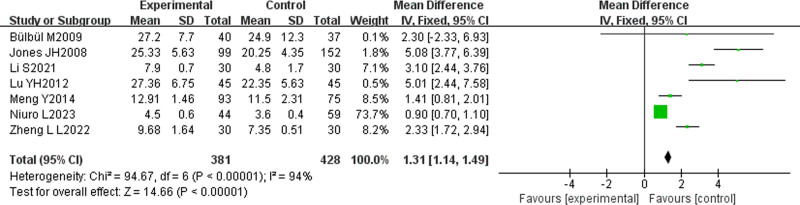
Meta-analysis results of body weight.

Seven studies in the included literature^[14,19–23,29]^ described the efficacy of L-T4 on weight development in children with CH, involving a total of 809 children, with 381 and 428 patients, respectively divided into in the treatment and control groups. Heterogeneity analysis I² = 94% > 50%, random-effects model was chosen, and META analysis showed a combined effect size MD = 1.31, 95% CI (1.14, 1.49), Z = 14.66, *P* < .00001, indicating that children in the experimental group had a significantly better weight development than the control group, with statistically significant difference. As shown in Figure 7.

#### 3.2.4. Meta-analysis results on head circumference development

**Figure 8.**
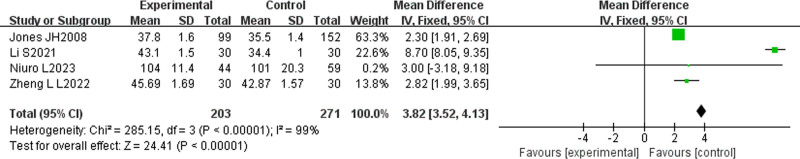
Meta-analysis results of head circumference.

Four studies in the included literature^[19,20,23,29]^ described the efficacy of L-T4 on head circumference development in children with CH, involving 474 children, with 203 and 271 patients in the treatment and control groups, respectively. Heterogeneity analysis I² > 50%, random-effects model was chosen, and META analysis showed a combined effect size MD = 3.83, 95% CI (3.52, 4.13), Z = 24.41, *P* < .00001, indicating that the head circumference development of children in the experimental group was significantly better than that of the control group, and the difference was statistically significant. As shown in Figure 8.

#### 3.2.5. Meta-analysis of results on bone age development

**Figure 9.**
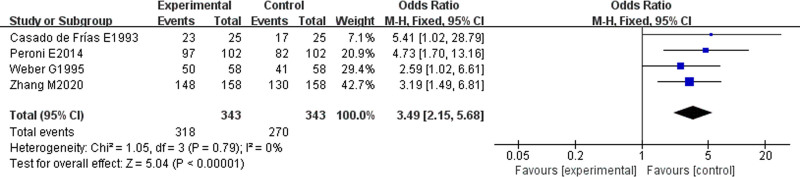
Meta-analysis results of bone age.

Four studies in the included literature^[15,24,27,28]^ described the efficacy of L-T4 on IQ development in children with CH, involving a total of 686 children, with 343 and 343 patients in the treatment and control groups respectively. Heterogeneity analysis I² = 0%, fixed-effects model was selected, and META analysis showed a combined effect size OR = 3.49, 95% CI (2.15, 5.68), Z = 5.04, *P* < .00001, indicating that children in the test group had a significantly better development of bone age than those in the control group, and the difference was statistically significant. As shown in Figure 9.

## 4. Discussion

CH is an endocrine disorder that manifests during the neonatal period or infancy and is primarily characterized by impaired synthesis or secretion of THs.^[30]^ Thyroid hormones play a crucial role in the growth and development of the human body, influencing the development and functionality of several organ systems, with a particular emphasis on the maturation of the nervous system.^[31]^ Therefore, TH insufficiency not only impacts children’s physical development indicators such as height and weight, but also may trigger long-term and irreversible damage to their intelligence, cognition and behavior.^[32]^ L-T4, serving as a therapeutic drug substitute for exogenous TH, occupies a central position in the treatment of CH.^[33]^ It replenishes the deficient TH in the child’s body, thereby ameliorating or correcting a range of physiological and pathological changes induced by hormone deficiency.^[34]^ In neonates and infants, the primary goal of L-T4 therapy is to rapidly increase the circulating levels of TH to normalize serum TSH,^[35]^ and once TSH levels are normalized, the L-T4 dose should be maintained at the same level, with regular monitoring to ensure TSH remains within the age-specific reference range.^[36]^ The first clinical and biochemical follow-up assessments occur within 1 to 2 weeks of initiating treatment, and the frequency of assessments is subsequently adjusted based on the children’s age until growth is completed.^[37]^ A diagnostic reassessment of thyroid function should be conducted after 6 months of life to determine whether the CH is permanent.^[38]^ For children with a confirmed diagnosis, L-T4 therapy should be continued, and the dose should be adjusted according to changes in thyroid function.^[39]^ As studies continue to progress, conclusions regarding the efficacy of L-T4 on the growth and development of children with CH have become inconsistent, stemming from differences and variations in sample size, age of the children, treatment dosage, duration of follow-up, and other relevant factors. These differentiators have led to inconsistencies in the results of the studies, which in turn have posed a challenge for clinical practice.^[40]^

TH is essential for the neuro-development of neonatal and infant, particularly in the development of the cerebral cortex, neuronal proliferation and migration, synapse formation, and myelination.^[41]^ It has an impact on the proliferation and differentiation of neural precursor cells as well as neuronal migration from the ventricular zone to the cortex. During migration, TH influences the expression of cell adhesion molecules and cell cycle-related proteins by making adjustments to the correct localization of neurons.^[42]^ TH fosters the formation and function of neural networks by promoting the development of presynaptic and postsynaptic structures, thus, increasing synaptic density.^[43]^ TH plays an important role in the maturation of oligodendrocytes and the formation of myelin sheaths, which accelerates nerve conduction and is indispensable for the development of cognitive functions.^[44]^ Children with CH experience significant impairments in the development of cognitive function due to TH deficiencies that are affected in several of these areas, resulting in limited IQ development. The results of the meta-analysis presented in this paper indicated that children in the L-T4-treated experimental group was exhibited significantly better IQ development than that of the control group, with MD = 8.38, 95% CI (6.89, 9.87), Z = 11.02, *P* < .00001, and that L-T4 was effective in increasing the TH levels in these children. All the included studies reported that after L-T4 treatment, total triiodothyronine, free triiodothyronine, total blood tetraiodothyronine (total thyroxine), free tetraiodothyronine (free thyroxine), and TSH levels were rapidly returned to normal values. Total triiodothyronine and free triiodothyronine are the active forms of THs, which are essential for promoting the development and maturation of the nervous system; while total blood tetraiodothyronine and free tetraiodothyronine are the major storage and transport forms of THs, which reflects the functionality of thyroid gland.^[45]^ TSH is a hormone secreted by the pituitary gland that stimulates the thyroid gland to secrete THs.^[46]^ L-T4, as an exogenous TH replacement therapy, can directly replenish the missing THs in children. Additionally, L-T4 treatment can improve the speed of nerve conduction and neuronal activity, promote neuronal proliferation and differentiation, affect the formation of synapses and neurotransmitter release, as well as promote myelin release, and formation of myelin sheaths.^[47]^ Consequently, L-T4 treatment can increase the functional activity of the children’s nervous system, improving their brain’s information processing ability, and learning efficiency.^[48]^

In infants, CH may lead to feeding difficulties and growth retardation; while in older children and adolescents, CH may lead to sluggish growth or delayed pubertal development.^[49]^ Untreated CH may lead to mental retardation and short stature, placing a serious burden on families and society.^[50]^ The META analysis conducted in this study revealed that L-T4 treatment could effectively promote the physical development of children, and the height [MD = 6.71, 95% CI (6.08, 7.35)], weight [MD = 1.31, 95% CI (1.14, 1.49)], head circumference [MD = 3.83, 95% CI (3.52, 4.13)], and bone age of the test group [OR = 3.49, 95% CI (2.15, 5.68)] were significantly better than those of the control group. L-T4 supplemented the TH deficiency, elevates the body’s metabolic rate, and stimulates protein synthesis, cell proliferation, and bone growth, thereby facilitating the physical development of the affected children. Among children with CH, TH deficiency slower, the bone development, resulting in an obvious disparity between bone age and actual age. L-T4 treatment can amend the TH deficiency, accelerate the rate of bone development, and normalize bone age, which contributes to the enhancement of normal body development. Thyroid hormone has an important effect on protein synthesis, and L-T4 treatment suit the remedy to the case that it can enhances the synthesis of skeletal muscle, thus promoting muscle development and weight gain in children.^[51]^ Furthermore, there is an interaction between TH and growth hormone, and TH deficiency may impair growth hormone secretion and growth hormone receptor sensitivity, but L-T4 treatment can restore TH levels, normalizing growth hormone levels and then promoting normal body growth and development.^[52]^ Children with CH are often associated with a loss of appetite and poor digestion and absorption, and L-T4 treatment can improve thyroid function that increases appetite and promotes the absorption of nutrients, which can help the increase of weight and head circumference expansion of these children.^[53]^

## 5. Conclusion

This meta-analysis clearly demonstrates the significant efficacy of L-T4 in the treatment of growth and development of children with CH. As an effective therapeutic replacement drug, L-T4 successfully promotes the physical and intellectual development of children by supplementing the missing THs in their bodies. The study showed that children in the L-T4 treatment group significantly outperformed those in the control group across various growth indicators, including IQ, height, weight, head circumference, and bone age. This result not only reflects the role of L-T4 in correcting TH levels, improving metabolism and energy supply, but also highlights its unique advantages in promoting bone and muscle development, refining nerve conduction, and cognitive function.

However, the present study harbors certain limitations. Firstly, although it has been shown that L-T4 treatment can promote the intellectual and physical development of children, the underlying mechanisms have not been further investigated. Secondly, the overall heterogeneity of this META analysis is relatively high, potentially attributed to variations in the methodologies employed in the experimental and control groups of the included articles, as well as the disparities in IQ assessment and other indicators. In conclusion, L-T4 suggests significant efficacy in the treatment of growth and development of children with CH and provides clinicians with a powerful therapeutic alternative. It is expected that more studies will be conducted in the future to further delve into mechanisms and optimization strategies of L-T4 in the treatment of children with CH, which will bring better treatment outcome and an enhanced life quality for these children.

## Author contributions

**Conceptualization:** Wenguan Liang.

**Data curation:** Wenguan Liang, Lin Tu.

**Formal analysis:** Wenguan Liang, Lin Tu.

**Funding acquisition:** Wenguan Liang.

**Investigation:** Wenguan Liang.

**Methodology:** Wenguan Liang, Lin Tu.

**Project administration:** Wenguan Liang, Lin Tu.

**Resources:** Wenguan Liang.

**Software:** Wenguan Liang.

**Supervision:** Wenguan Liang.

**Validation:** Wenguan Liang.

**Visualization:** Wenguan Liang.

**Writing – original draft:** Wenguan Liang, Lin Tu.

**Writing – review & editing:** Wenguan Liang, Lin Tu.
